# Nonparametric Method for Genomics-Based Prediction of Performance of Quantitative Traits Involving Epistasis in Plant Breeding

**DOI:** 10.1371/journal.pone.0050604

**Published:** 2012-11-30

**Authors:** Xiaochun Sun, Ping Ma, Rita H. Mumm

**Affiliations:** 1 Department of Crop Sciences and the Illinois Plant Breeding Center, University of Illinois at Urbana-Champaign, Urbana, Illinois, United States of America; 2 Department of Statistics; University of Illinois at Urbana-Champaign, Urbana, Illinois, United States of America; Cleveland Clinic Lerner Research Institute, United States of America

## Abstract

Genomic selection (GS) procedures have proven useful in estimating breeding value and predicting phenotype with genome-wide molecular marker information. However, issues of high dimensionality, multicollinearity, and the inability to deal effectively with epistasis can jeopardize accuracy and predictive ability. We, therefore, propose a new nonparametric method, pRKHS, which combines the features of supervised principal component analysis (SPCA) and reproducing kernel Hilbert spaces (RKHS) regression, with versions for traits with no/low epistasis, pRKHS-NE, to high epistasis, pRKHS-E. Instead of assigning a specific relationship to represent the underlying epistasis, the method maps genotype to phenotype in a nonparametric way, thus requiring fewer genetic assumptions. SPCA decreases the number of markers needed for prediction by filtering out low-signal markers with the optimal marker set determined by cross-validation. Principal components are computed from reduced marker matrix (called supervised principal components, SPC) and included in the smoothing spline ANOVA model as independent variables to fit the data. The new method was evaluated in comparison with current popular methods for practicing GS, specifically RR-BLUP, BayesA, BayesB, as well as a newer method by Crossa *et al.,* RKHS-M, using both simulated and real data. Results demonstrate that pRKHS generally delivers greater predictive ability, particularly when epistasis impacts trait expression. Beyond prediction, the new method also facilitates inferences about the extent to which epistasis influences trait expression.

## Introduction

The estimation of breeding values to facilitate choice of parents is a central problem in plant breeding. Furthermore, in terms of evaluating and identifying outstanding progeny, modern genotyping technologies make it possible to predict performance of new lines based on molecular marker or DNA sequence profile.

Fernando and Grossman [1] first demonstrated the utility of molecular marker data to estimate breeding values in livestock species. These were data involving very few markers. Due to increasingly developed genotyping and sequencing technologies, densely spaced genome-wide SNP (single nucleotide polymorphism) data, involving tens or hundreds of thousands of markers, are now available for a number of crops. The genome-wide markers can be used as ‘predictors’ to achieve high accuracy in estimating breeding values. However, problems like high dimensionality and multicollinearity emerge when the number of predictors is very large and exceeds the number of records. Therefore, statistical methods to effectively address those issues are urgently needed.

Meuwissen *et al.* [2] proposed a procedure called Genomic Selection (GS), which uses genome-wide markers to estimate breeding values. Through linear regression of phenotypes on genome-wide markers, this method can model high-dimensional predictors. Then, a shrinkage method can be applied to effectively ‘shrink’ the effect of multicollinearity and to provide stable parameter estimates [3]. Utilizing these techniques, this approach can generate information about genomic regions that may affect the trait of interest. More recently, other shrinkage methods have been developed to estimate breeding values [4], [5]. These methods are primarily based on linear models, which are easy to interpret and able to fit to the data without overfitting. However, the relationship between breeding value and genetic markers is likely to be more complex than a simple linear relationship, particularly when large numbers of SNPs are fitted simultaneously in the model. While epistasis is recognized as an important source of genetic variation [6], strong genetic assumptions are needed to statistically decompose epistatic variance in those linear models [7], *e.g.* additive by additive epistasis. Furthermore, the fact that biological basis of epistasis is not well understood makes accommodation for it in the genetic model even more difficult. To address these issues, model-free or so-called nonparametric methods which side-step linearity and require fewer genetic assumptions have gained more and more attention [8]–[10].

Gianola *et al.* [10] and Gianola and van Kaam [8] first proposed reproducing kernel Hilbert spaces (RKHS) regression for estimating breeding values with genomic data and capturing epistatic interactions. The fundamental idea of the RKHS methods is to replace the original marker values with nonlinear transformed markers through so-called ‘basis functions’. After transformation, a new space of predictors is formed and can be used in regression. In reproducing kernel Hilbert spaces, the basis functions are reproducing kernels, which vary according to different inner products defined in the RKHS. Gianola and van Kaam [8] proposed using a multivariate Gaussian kernel suggested by Mallick *et al.* [11] as a reproducing kernel. However, the multivariate Gaussian kernel assigns equal weights to all predictors. Consequently, one cannot determine which predictors are important and which not by using RKHS regression. Thus, model selection and model building by eliminating unimportant predictors are impossible in this simple framework. Intrinsically, the method becomes a mere function approximation method rather than a statistical modeling method. Besides RKHS methods, Bennewitz *et al.* [12] also explored the use of a kernel method which originated from Nadaraya-Watson kernel regression [13], [14] to estimate breeding values. However, the kernel methods incur substantial bias when applied to high dimensional regression with interactions [15].

To overcome the shortcomings of the aforementioned approaches, in this paper, we introduce a smoothing spline ANOVA method in RKHS [16]–[18]. The distinguished feature of the method is decomposition of the multivariate nonparametric function into main effects and interactions, analogous to classic ANOVA in linear models. This gives rise to straightforward interpretation of each component, which distinguishes it from function approximation in Gianola and van Kaam [8]. Assigning different weights to main effects and interactions by some data-driven approach, one can easily conduct model diagnostics, model selection, and model building, However, with tens of thousands of markers in the model, the fitting of RKHS models is computationally expensive, even infeasible [17]. The resulting algorithm is unstable and error-prone. One solution would be to bring down the dimensionality of predictors through usage of a dimension reduction method such as principal component analysis [19]. Alternatively, prediction accuracy may be increased by filtering out ‘noisy’ markers, an approach supported by results from Meuwissen *et al.* [2] demonstrating that GS method BayesB which allows certain markers to have no associations with phenotypes had high predictive ability. For the latter, Macciotta *et al.* [20] and Schulz-Streeck *et al.* [21] assigned a *p*-value to each marker through univariate linear regression and used an empirical threshold to remove markers without strong signal. And Long *et al.* [22] used two steps called “filter” and “wrapper” to select SNPs. Supervised principal component analysis (SPCA) [23] offers both dimension reduction and background noise reduction and serves to supplement RKHS regression.

In this study, we devise and evaluate a two-step method (pRKHS), combining SPCA and RKHS regression, to estimate breeding values and predict phenotypic performance of lines with or without pedigree relationships. In step one, we preselect genetic markers highly correlated with phenotype, and perform principal component analysis on the reduced marker subset. In step two, we use significant principal components as predictors in a smoothing spline ANOVA model to conduct the RKHS regression. The model is fitted using a penalized least squares method, where goodness-of-fit is measured by the least squares and model complexity is dictated by a penalty. The trade-off between goodness-of-fit and model complexity is controlled by smoothing parameters, which are selected by data-driven generalized cross-validation (GCV). The pRKHS method is developed in two versions: pRKHS-NE, which accounts for only additive effects, and pRKHS-E, which includes additive-by-additive interaction effects as well as additive effects in the model. The pRKHS versions are evaluated for predictive ability in simulated genetic scenarios and confirmed in real life scenarios for utility using actual data from corn and barley. The pRKHS versions are also compared in performance with popular shrinkage methods, specifically RR-BLUP, BayesA, BayesB [2] and the nonparametric method RKHS-M used by Crossa *et al.* [24].

## Materials and Methods

### Simulation

#### Mating scheme

The breeding scheme for maize line development outlined by Bernardo and Yu [25] was used in the simulation of a number of plant breeding scenarios. Specifically, two unrelated inbreds were crossed to produce an F_1_ population, from which N doubled haploid (DH) lines (Cycle 0) were generated and crossed to a common tester. Testcross performance data and genotypes of Cycle 0 lines were used to train the model. Based on Cycle 0 testcross phenotypes, N_sel_ lines were selected to randomly mate for two generations to produce N Cycle 1 lines. Genotypes of Cycle 1 lines were used to predict testcross phenotypes using fitted model. The marker data were coded as *z_ij = _*1, if *j*th marker locus in *i*th individual was homozygous for marker allele from parental Inbred 1, *z_ij_* = -1 if homozygous for marker allele from parental Inbred 2 and 0 if heterozygous. N and N_sel_ values were set as 144 and 8, respectively, according to Bernardo and Yu [25].

### Genome model

The genome model for simulation was constructed according to the published maize ISU–IBM genetic map, with a total of 1788 cM [26], with recombination computed using the Kosambi map function [27]. Markers were evenly spaced on the chromosome at 1 cM intervals. And 100 QTLs were randomly positioned across the genome. Both markers and QTLs were assumed to be bi-allelic. The genotypic value for *i*th individual was calculated according to [7]

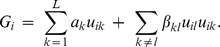



Design element 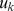 is defined according to the general two-allele model (G2A, [28] as.
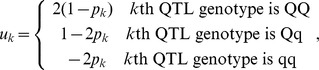
where *p_k_* is the allele frequency of Q in *k*th QTL. Parameter 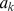 is *k*th QTL’s additive effect and 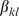 is the epistatic interaction effect between *k*th and *l*th QTL. In this case, 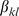 indicates additive by additive interaction. Furthermore, 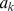
was sampled from geometric series 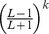 [25], [29], where *L* equals the total number of QTL positioned throughout the genome. The direction of effect was randomly assigned to each QTL, leading to random coupling and repulsion linkages. Epistatic effect 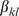 was sampled from gamma distribution



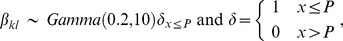
where δ is the indicator function which compares the random variable *x* generated from uniform distribution [0,1] with P. The extent of epistasis was specified by assigning the proportion (*P*) of total epistatic interactions with nonzero effect. Three levels of epistasis were considered: *P* = 0, 0.1 and 0.5, representing no, low, and high epistasis, respectively. The G2A model [28] was chosen to model QTL due to its orthogonal property, which links the genetic variance partition directly to the genetic effect partition. The genetic variance was therefore calculated from the sample variance of genotypic values. Random nongenetic effects were added to the genotypic values to generate phenotypic values in proportion to the heritability (*i.e.* four heritability levels were considered: 0.1, 0.2, 0.4 and 0.8).

### Real Data

To evaluate the predictive ability under real life scenarios, data reported by Crossa *et al*. [24] on 284 maize lines genotyped with 1148 and 1135 SNPs and phenotyped for anthesis-silking interval (ASI) and grain yield (GY), respectively, were utilized. In addition, three barley datasets generated from North Dakota State University two-rowed (N2) breeding program, with trial name of ‘Expt41_2007_Langdon’, ‘Expt41_2008_Langdon’ and ‘Exp41_2009_Langdon’ from The Hordeum Toolbox (http://wheat.pw.usda.gov/tht/) were utilized. Only entries with phenotypic observations for both grain yield (GYD) and plant height (PHT) from the same location were used to avoid confounding genotype with environment. Trials of year 2007 and 2008 contained different sets of 96 lines while 2009 trial had 57 lines, for a total of 249 unique lines across the three years. Lines of one year were independent from those of others. There were 2161 SNPs for the 2007 dataset, 2029 SNPs for the 2008 dataset, and 1842 SNPs for the 2009 dataset, among which 1641 markers were shared among three years. After filtering out markers with minor allele frequency (*i.e*. smaller than 0.05), 1511 SNPs were retained for barley data.

SNPs were bi-allelic and the dummy variable for marker data is defined as *z_ij = _*1 for A_1_A_1_, *z_ij = _*0 for A_1_A_2_ and *z_ij_* = −1 for A_2_A_2_. For SNP data from BarleyCAP, genotypes ‘1∶1’, ‘2∶2’, and ‘1∶2’ were considered as A_1_A_1_, A_2_A_2_, and A_1_A_2_, respectively. Although the type of marker data is discrete, it is treated as continuous vector of covariates. Missing markers were imputed by averaging marker scores across all lines of that marker. Two missing phenotypes from 2007 and 2008 data were imputed using k-nearest-neighbor (KNN) algorithm [30].

### Statistical Methods

#### pRKHS-E & pRKHS-NE

Features from SPCA and RKHS regression were combined to develop the new method, pRHKS. First, SPCA was applied to reduce the high dimensionality represented by the markers and to decrease ‘noise’. Steps to apply SPCA included:

- Computing the regression coefficient for each marker on a single marker basis,
- Ranking markers by the absolute value of their regression coefficients and selecting a defined number of the top ranked markers to form a marker subset (MS) with which to construct the reduced data matrix,
- Performing principal component analysis using the reduced data matrix to generate resulting PCs, referred to as supervised principal components (SPCs).

A series of SPCs *i.e.* explaining 55%,60%, 65%,70%, 75%,80%, and 85% of the data matrix variance were then considered as independent variables to fit a smoothing spline ANOVA model in reproducing kernel Hilbert spaces [17]. Results led to a determination that ∼70% (±10%) was an optimal threshold of PC variation explained by the model to achieve high prediction accuracy.

Two versions of the new method (pRKHS-NE and pRKHS-E) were proposed to account for various levels of epistasis.

1. pRKHS-NE: All selected SPCs were included in the model as main effects. No interactions were included.
2. pRKHS-E: Main effects and two-way, additive-by-additive interactions were included in the model, specifying the level of epistasis. For example, when epistasis was specified as 0.1, then 10% of the epistasis interaction effects were considered to be nonzero. To prevent high dimensionality, each variable and their pair-wise interactions were tested for significance and selected using non-parametric model diagnostics tools, *i.e.* cosine value (CoV) [17], which corresponds roughly to F-statistics in a parametric regression model. Main effects with CoV larger than 0.05 were retained in the model. To determine the tolerance for various different levels of epistatic interactions, a series of CoV *i.e.* 0.3, 0.25 and 0.2 were considered.

The nonparametric model pRKHS is written as.
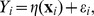
where *Y_i_* is the phenotype of *i*th individual, 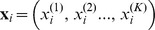 is the vector of *k* SPCs 
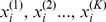
of *i*th individual, 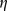 is some unknown K-variate function relating SPCs and phenotype, and 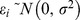 is error term for *i*th individual. We estimate 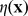 in a functional space 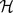 using a penalized least squares,



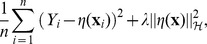
where the first term measures the goodness of fit, and 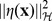 quantifies the smoothness of η, and λ is a smoothing parameter balancing the trade-off between the two conflicting goals. Full details of the derived model are described in Appendix S1.

#### Comparison methods

pRKHS-E and pRKHS-NE were compared to three shrinkage methods: RR-BLUP, BayesA and BayesB [2]. The general model was written as 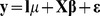, where 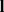 is a vector filled with ones, 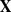 is marker data matrix, 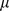 is the fixed grand mean, 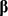 is the vector of marker effects, and 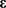 is the vector of random residuals. A Gaussian prior was assigned to 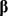 and 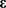, with 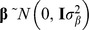 and 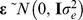. RR-BLUP was implemented in a Bayesian framework; it assigns a common variance to all marker effects, whereas BayesA and BayesB assigns different variances to different markers. BayesB was modified in this study to include π, the proportion of markers having no genetic variances, as another parameter in the model and assigned it a uniform [0,1] prior instead of arbitrary setting [31]. Variances 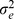 and 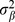 were assigned a scaled inverse chi-square distribution with scale
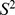
and degree of freedom 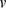.

Besides shrinkage methods, pRKHS was also compared to the RKHS-M model mentioned in work by Crossa *et al.* [24]. The model was 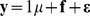, where 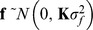. 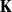 was the kernel matrix whose 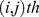 entry equaled 
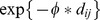
, where 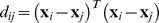 measured similarity between *i*th and *j*th individuals, where 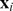 is a vector of marker scores of *i*th individual and bandwidth parameter 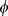 was chosen as 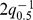, where 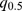 was the sample median of 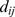. And 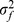 was also assigned a scaled inverse chi-square distribution with scale 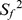 and degree of freedom 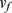.

### Data Analysis

The smoothing spline ANOVA model in pRKHS-E and pRKHS-NE was fitted using the *ssanova* function in “gss” package available in R [32]. Default arguments of *ssanova* function were used, *i.e.* argument “*method*” was set to “*v*” to let smoothness parameter *λ* be selected by GCV and “*type*” was set to “*cubic*” to use a cubic smoothing spline. RR-BLUP, BayesA, and BayesB were coded using C++, among which 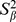 = 4.23, 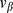 = 0.05 and 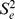 = 1, 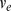 = 1. The RKHS method was implemented using the program provided by Crossa *et al.* [24], with 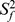 = 4 and 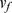 = 1. Gibbs sampler was implemented with 3 chains and 10,000 iterations for each chain to update conditional posterior distributions. The first 1,000 samples of each chain were discarded as burn-in and later thinned by 10. Convergence was checked by inspection of trace plots and Gelman-Rubin plots of error variance using “coda” package in R [33]. Samples from three chains were combined to estimate posterior means. All analyses were run on an Ubuntu Server with 2.8 GHz CPU and 16 GB memory.

In simulation scenarios, ten-fold CV in Cycle 0 (C0) was used to determine the best MS and CoV for pRKHS-E(NE). Pearson correlation coefficients between estimated breeding value (EBV) and true breeding value (TBV) (r_EBV:TBV_), and between EBV and phenotype (PHE) (r_EBV:PHE_) were calculated and averaged across thirty replicated simulations.

Since TBV will never be observed in real cases, the criterion to select MS and CoV was based on r_EBV:PHE_ rather than r_EBV:TBV_. Given the highest r_EBV:PHE_ in C0, optimum MS and CoV were determined and used to estimate breeding values and phenotypes in Cycle 1 (C1). Across a series of MS (*i.e.* from 500 to all markers), percent of variation and number of influential markers whose loadings >0.8*the maximum loading were extracted from top three SPCs, and number of SPC interactions with CoV being 0.2, 0.25 and 0.3 were also recorded.

In real data applications, predicted maize ASI and GY values were based on five-fold CV since only one set of data was available. With barley, r_EBV:PHE_ was computed to measure predictive ability. Expt41_2007_Langdon barley data were used for ten-fold CV to select the optimum MS and CoV for pRKHS-E(NE), which were further used to predict phenotypes of trial Expt41_2008_Langdon and Expt41_2009_Langdon. Model fitting for all real data was repeated five times and results were reported as a mean of five. The R code for computing pRKHS EBVs and correlations with phenotype using the three barley datasets generated by North Dakota State barley breeding program (Expt41_2007_Langdon, Expt41_2008_Langdon and Expt41_2009_Langdon accessed through The Hordeum Toolbox http://wheat.pw.usda.gov/tht/) is provided in the supplemental materials (R Code S1).

## Results

### Simulation Results

Twelve different scenarios were considered in this study to facilitate comparison of methods given various levels of heritability and epistasis (Tables 1, 2, and 3). Pearson correlation coefficients for EBV: TBV (r_EBV:TBV_) and for EBV: PHE (r_EBV:PHE_) were calculated (Tables 1, 2 and 3). Both ten-fold CV in C0 and prediction in C1 were used to assess the predictability of the statistical methods.

**Table 1:** For scenarios with no epistasis, Pearson correlation coefficients between estimated breeding value and true breeding value (r_EBV:TBV_) or phenotype (r_EBV:PHE_) obtained through ten-fold cross-validation with Cycle 0 (C0) and prediction of Cycle 1(C1), implemented for simulated traits with heritability of 0.1, 0.2, 0.4, 0.8, via the various statistical methods.

Heritability	C0/C1	Methods	r_EBV:TBV_ ± SE	r_EBV:PHE_ ± SE
h^2^ = 0.1	C0	RR-BLUP	0.474±0.015	0.174±0.019
	C0	BayesA	0.451±0.016	0.170±0.023
	C0	BayesB	0.475±0.015	0.180±0.020
	C0	RKHS-M	0.350±0.021	0.103±0.018
	C0	pRKHS-E	0.422±0.019	0.189±0.005
	C0	pRKHS-NE	0.480±0.016	0.192±0.013
	C1	RR-BLUP	0.329±0.017	0.127±0.018
	C1	BayesA	0.307±0.020	0.124±0.017
	C1	BayesB	0.338±0.017	0.134±0.018
	C1	RKHS-M	0.252±0.014	0.066±0.016
	C1	pRKHS-E	0.342±0.023	0.121±0.026
	C1	pRKHS-NE	0.382±0.016	0.155±0.019
h^2^ = 0.2	C0	RR-BLUP	0.572±0.019	0.235±0.018
	C0	BayesA	0.568±0.015	0.230±0.014
	C0	BayesB	0.582±0.018	0.244±0.010
	C0	RKHS-M	0.442±0.012	0.179±0.018
	C0	pRKHS-E	0.494±0.011	0.248±0.013
	C0	pRKHS-NE	0.599±0.018	0.254±0.010
	C1	RR-BLUP	0.470±0.019	0.289±0.015
	C1	BayesA	0.431±0.010	0.265±0.011
	C1	BayesB	0.479±0.020	0.298±0.013
	C1	RKHS-M	0.363±0.018	0.235±0.019
	C1	pRKHS-E	0.341±0.018	0.180±0.011
	C1	pRKHS-NE	0.450±0.019	0.257±0.015
h^2^ = 0.4	C0	RR-BLUP	0.785±0.014	0.421±0.018
	C0	BayesA	0.697±0.017	0.354±0.016
	C0	BayesB	0.799±0.016	0.427±0.015
	C0	RKHS-M	0.614±0.017	0.352±0.012
	C0	pRKHS-E	0.756±0.017	0.395±0.011
	C0	pRKHS-NE	0.789±0.020	0.388±0.014
	C1	RR-BLUP	0.614±0.013	0.425±0.011
	C1	BayesA	0.529±0.013	0.361±0.015
	C1	BayesB	0.622±0.013	0.433±0.020
	C1	RKHS-M	0.535±0.022	0.384±0.023
	C1	pRKHS-E	0.513±0.016	0.381±0.016
	C1	pRKHS-NE	0.574±0.018	0.402±0.017
h^2^ = 0.8	C0	RR-BLUP	0.827±0.009	0.729±0.006
	C0	BayesA	0.763±0.012	0.673±0.004
	C0	BayesB	0.831±0.009	0.735±0.008
	C0	RKHS-M	0.768±0.011	0.698±0.009
	C0	pRKHS-E	0.678±0.016	0.686±0.012
	C0	pRKHS-NE	0.815±0.014	0.675±0.012
	C1	RR-BLUP	0.744±0.012	0.674±0.014
	C1	BayesA	0.664±0.021	0.601±0.022
	C1	BayesB	0.752±0.011	0.682±0.013
	C1	RKHS-M	0.675±0.010	0.620±0.010
	C1	pRKHS-E	0.633±0.008	0.571±0.011
	C1	pRKHS-NE	0.734±0.010	0.613±0.009

Average correlations ± SE were obtained from thirty replications of each simulation.

**Table 2:** For scenarios with a low level of epistasis (10% of the epistasis interaction effects are nonzero), Pearson correlation coefficients between estimated breeding value and true breeding value (r_EBV:TBV_) or phenotype (r_EBV:PHE_) obtained through ten-fold cross-validation with Cycle 0 (C0) and prediction of Cycle 1 (C1), implemented for simulated traits with heritability of 0.1, 0.2, 0.4, 0.8, via the various statistical methods.

Heritability	C0/C1	Methods	r_EBV:TBV_ ± SE	r_EBV:PHE_ ± SE
h^2^ = 0.1	C0	RR-BLUP	0.418±0.015	0.144±0.009
	C0	BayesA	0.402±0.015	0.134±0.008
	C0	BayesB	0.421±0.014	0.143±0.009
	C0	RKHS-M	0.257±0.012	0.089±0.008
	C0	pRKHS-E	0.433±0.012	0.169±0.018
	C0	pRKHS-NE	0.419±0.015	0.142±0.015
	C1	RR-BLUP	0.369±0.017	0.164±0.010
	C1	BayesA	0.340±0.019	0.153±0.011
	C1	BayesB	0.367±0.018	0.163±0.010
	C1	RKHS-M	0.258±0.018	0.100±0.008
	C1	pRKHS-E	0.394±0.021	0.168±0.005
	C1	pRKHS-NE	0.358±0.017	0.159±0.006
h^2^ = 0.2	C0	RR-BLUP	0.535±0.011	0.228±0.019
	C0	BayesA	0.518±0.008	0.234±0.016
	C0	BayesB	0.536±0.011	0.235±0.018
	C0	RKHS-M	0.435±0.014	0.186±0.016
	C0	pRKHS-E	0.542±0.010	0.237±0.015
	C0	pRKHS-NE	0.540±0.010	0.245±0.019
	C1	RR-BLUP	0.512±0.015	0.313±0.016
	C1	BayesA	0.479±0.014	0.267±0.014
	C1	BayesB	0.514±0.015	0.315±0.016
	C1	RKHS-M	0.413±0.010	0.234±0.015
	C1	pRKHS-E	0.484±0.014	0.336±0.006
	C1	pRKHS-NE	0.481±0.014	0.326±0.011
h^2^ = 0.4	C0	RR-BLUP	0.688±0.007	0.444±0.008
	C0	BayesA	0.632±0.009	0.421±0.003
	C0	BayesB	0.687±0.006	0.438±0.008
	C0	RKHS-M	0.569±0.011	0.358±0.018
	C0	pRKHS-E	0.696±0.009	0.448±0.008
	C0	pRKHS-NE	0.681±0.011	0.434±0.008
	C1	RR-BLUP	0.606±0.017	0.377±0.015
	C1	BayesA	0.535±0.008	0.327±0.010
	C1	BayesB	0.600±0.020	0.372±0.017
	C1	RKHS-M	0.503±0.013	0.320±0.011
	C1	pRKHS-E	0.605±0.021	0.372±0.020
	C1	pRKHS-NE	0.615±0.016	0.384±0.015
h^2^ = 0.8	C0	RR-BLUP	0.802±0.001	0.692±0.002
	C0	BayesA	0.734±0.003	0.633±0.006
	C0	BayesB	0.816±0.004	0.699±0.006
	C0	RKHS-M	0.776±0.004	0.698±0.007
	C0	pRKHS-E	0.809±0.012	0.694±0.012
	C0	pRKHS-NE	0.821±0.007	0.701±0.010
	C1	RR-BLUP	0.770±0.013	0.690±0.012
	C1	BayesA	0.710±0.012	0.634±0.011
	C1	BayesB	0.787±0.013	0.705±0.011
	C1	RKHS-M	0.751±0.014	0.689±0.014
	C1	pRKHS-E	0.775±0.013	0.693±0.010
	C1	pRKHS-NE	0.797±0.014	0.712±0.012

Average correlations ± SE were obtained from thirty replications of each simulation.

**Table 3:** For scenarios with a moderate level of epistasis (50% of the epistasis interaction effects are nonzero), Pearson correlation coefficients between estimated breeding value and true breeding value (r_EBV:TBV_) or phenotype (r_EBV:PHE_) obtained through ten-fold cross-validation with Cycle 0 (C0) and prediction of Cycle 1 (C1), implemented for simulated traits with heritability of 0.1, 0.2, 0.4, 0.8, via the various statistical methods.

Heritability	C0/C1	Methods	r_EBV:TBV_ ± SE	r_EBV:PHE_ ± SE
h^2^ = 0.1	C0	RR-BLUP	0.372±0.021	0.175±0.022
	C0	BayesA	0.363±0.020	0.158±0.023
	C0	BayesB	0.336±0.016	0.141±0.015
	C0	RKHS-M	0.173±0.018	0.119±0.013
	C0	pRKHS-E	0.382±0.020	0.203±0.020
	C0	pRKHS-NE	0.363±0.018	0.171±0.021
	C1	RR-BLUP	0.309±0.013	0.182±0.011
	C1	BayesA	0.327±0.019	0.192±0.009
	C1	BayesB	0.298±0.019	0.188±0.010
	C1	RKHS-M	0.157±0.015	0.139±0.008
	C1	pRKHS-E	0.328±0.013	0.194±0.012
	C1	pRKHS-NE	0.298±0.010	0.176±0.011
h^2^ = 0.2	C0	RR-BLUP	0.487±0.022	0.172±0.020
	C0	BayesA	0.444±0.022	0.175±0.017
	C0	BayesB	0.507±0.024	0.184±0.025
	C0	RKHS-M	0.331±0.026	0.192±0.024
	C0	pRKHS-E	0.512±0.030	0.254±0.023
	C0	pRKHS-NE	0.492±0.024	0.230±0.021
	C1	RR-BLUP	0.416±0.020	0.282±0.011
	C1	BayesA	0.408±0.017	0.256±0.010
	C1	BayesB	0.416±0.008	0.299±0.011
	C1	RKHS-M	0.295±0.011	0.214±0.005
	C1	pRKHS-E	0.441±0.018	0.303±0.010
	C1	pRKHS-NE	0.435±0.014	0.286±0.008
h^2^ = 0.4	C0	RR-BLUP	0.526±0.016	0.263±0.015
	C0	BayesA	0.520±0.015	0.261±0.021
	C0	BayesB	0.557±0.017	0.300±0.019
	C0	RKHS-M	0.427±0.017	0.306±0.021
	C0	pRKHS-E	0.603±0.016	0.347±0.031
	C0	pRKHS-NE	0.551±0.018	0.333±0.023
	C1	RR-BLUP	0.504±0.022	0.311±0.018
	C1	BayesA	0.462±0.017	0.285±0.014
	C1	BayesB	0.511±0.021	0.315±0.017
	C1	RKHS-M	0.347±0.021	0.267±0.014
	C1	pRKHS-E	0.525±0.016	0.390±0.015
	C1	pRKHS-NE	0.463±0.014	0.344±0.014
h^2^ = 0.8	C0	RR-BLUP	0.680±0.009	0.407±0.007
	C0	BayesA	0.599±0.008	0.324±0.009
	C0	BayesB	0.697±0.011	0.420±0.008
	C0	RKHS-M	0.584±0.011	0.561±0.012
	C0	pRKHS-E	0.706±0.008	0.535±0.001
	C0	pRKHS-NE	0.660±0.009	0.480±0.010
	C1	RR-BLUP	0.612±0.013	0.298±0.029
	C1	BayesA	0.596±0.014	0.283±0.031
	C1	BayesB	0.637±0.023	0.320±0.028
	C1	RKHS-M	0.475±0.022	0.308±0.053
	C1	pRKHS-E	0.638±0.017	0.418±0.046
	C1	pRKHS-NE	0.618±0.020	0.281±0.036

Average correlations ± SE were obtained from thirty replications of simulation.

For scenarios with no epistasis, BayesB generally outperformed other methods in predictive ability (Table 1). BayesB provided the highest correlation between EBV and TBV in five out of eight cases, except in three cases at low heritability levels (h^2^ = 0.1 and 0.2), where pRKHS-NE outperformed or at least had comparable results with BayesB. The values of r_EBV:TBV_ for pRKHS-NE were consistently higher than those for pRKHS-E across both C0 and C1, in keeping with the scenario of no epistasis. In three cases where pRKHS-NE outperformed BayesB for r_EBV:TBV_, it also provided higher correlations between EBV and PHE than BayesB. In no instances did RR-BLUP, BayesA, or RKHS-M provide the highest correlations for TBV or PHE.

For scenarios with epistasis at a low level, the pRKHS method outperformed other methods in predictive ability; particularly in predicting PHE, the pRKHS method provided the highest correlation in all eight cases of C0 and C1 (Table 2). The values of pRKHS-E for r_EBV:TBV_ were marginally higher than those for pRKHS-NE in five out of eight cases of C0 and C1. The pRKHS method provided highest values for r_EBV:TBV_ in seven out of eight cases of C0 and C1, with BayesB providing the highest values in C1 case at h^2^ = 0.2. For the correlation with PHE, pRKHS-E exceeded pRKHS-NE in three out of four heritability scenarios (h^2^ = 0.1, 0.2, and 0.4) and performed below pRKHS-NE at h^2^ = 0.8. In no instances did RR-BLUP, BayesA, or RKHS-M provide the highest correlations for either TBV or PHE.

For scenarios with high epistasis, the pRKHS method, particularly pRKHS-E, outperformed other methods in predictive ability (Table 3). In all cases of C0 and C1 across all heritabilities, pRKHS-E provided the highest correlations for both TBV and PHE. Among four C0 cases, the magnitude of the values of r_EBV:TBV_ of BayesB and pRKHS-NE had decreased the most from the corresponding value in low epistasis scenario at heritability of 0.1 and 0.2, respectively. And RKHS-M experienced the most loss of accuracy at heritability of 0.4 and 0.8. In contrast, pRKHS-E showed the least amount of loss in accuracy based on change of r_EBV:TBV_ in all four C0 cases.

The advantage of marker-based selection (MBS) over phenotypic selection (PS) can be quantified by comparing r_EBV:TBV_ in C1 cases to accuracy of PS, defined as the correlation between mid-parent and offspring and measured by taking square root of half of the narrow-sense heritability [34]. For heritabilities of 0.1, 0.2, 0.4 and 0.8, the accuracy of PS was estimated as 0.224, 0.316, 0.447, and 0.632, respectively. For the scenarios with no and low epistasis (Tables 1 and 2), all methods outperformed PS at all four heritabilities, particularly at low heritability (h^2^ = 0.1 and 0.2). For the scenarios with high epistasis (Table 3), pRKHS-E and BayesB outperformed PS in all cases, whereas pRKHS-NE and RR-BLUP outperformed PS only at low to moderate heritabilities (h^2^ = 0.1, 0.2 and 0.4). RKHS-M did not outperform PS at any of the heritability levels.

**Figure 1:**
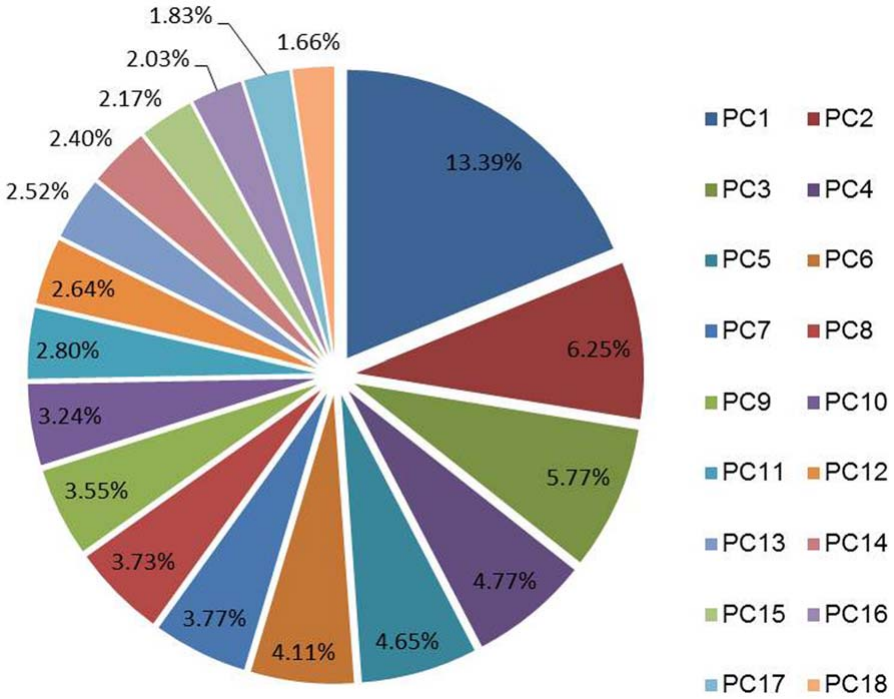
Mean percentage of variation (across the 12 simulation scenarios) explained by the top 18 SPCs with pRHKS, which together explain 70% of the total variation.

Table 4 exhibits the range of values for the percentage of variation explained by the first three SPCs, the number of markers included in each of these SPCs, and the number of SPC interactions observed when the cosine threshold for selecting SPC interactions was >0.2, >0.25, and >0.3, respectively, given the series of marker subsets (from as low as 500 markers to all, *i.e.* 1798 markers) used across 12 simulation scenarios. With low marker density, *i.e.* 500 markers, the first, second and third SPCs explained up to 18.7%, 11.8% and 9.9%, respectively, of the marker variation. Utilizing the full marker data set, the top three SPCs only explained as low as 9.2%, 5.5% and 5.1% of the variation (Table 4). Averaging across all scenarios, the first three SPC accounted for 25.4% of the marker variation and 18 SPCs were needed to explain 70% of the marker variation (Figure 1). The number of influential markers included in the first three SPCs, namely M_P1_, M_P2_ and M_P3_, varied according to the number of markers used in the model. Using all 1798 markers in simulations, the 1^st^, 2^nd^ and 3^rd^ SPCs included a maximum of 137, 111, and 103 influential markers, respectively; using the smallest subset of markers *i.e.* 500 markers, the 1^st^, 2^nd^, and 3^rd^ SPCs included as few as 61, 52, and 37 influential markers, respectively (Table 4). The number of SPC interactions was determined by the MS and CoV. High MS or low CoV generate a relatively large number of SPC interactions.

**Table 4:** For each scenario with pRKHS, the percent of the total variation explained by top three SPCs (%P1, %P2 and %P3), the number of influential markers (M_P1_, M_P2_ and M_P3_) included in the respective SPCs, and number of SPC interactions at three given cosine thresholds.

Scenarios	%P1	%P2	%P3	M_P1_	M_P2_	M_P3_	# of SPC interactions		
							>0.2	>0.25	>0.3
**h^2^ = 0.1, E = 0**	10.4–15.1	5.8–11.1	5.3–9.0	83–127	61–104	43–86	5–12	1–5	0–3
**h^2^ = 0.2, E = 0**	12.2–17.7	5.5–10.8	5.2–8.1	124–136	59–71	56–85	3–11	1–6	0–4
**h^2^ = 0.4, E = 0**	9.3–14.9	6.8–11.7	5.9–9.9	67–111	59–90	56–96	4–16	1–6	0–3
**h^2^ = 0.8, E = 0**	10.4–15.3	5.8–11.0	5.3–9.1	76–124	53–89	48–87	5–20	1–7	0–1
**h^2^ = 0.1, E = 0.1**	11.1–17.7	6.1–9.7	5.4–8.4	105–130	55–98	50–92	4–16	1–5	0–3
**h^2^ = 0.2, E = 0.1**	11.9–16.5	5.6–11.8	5.1–8.2	110–125	66–85	43–88	4–12	2–7	1–4
**h^2^ = 0.4, E = 0.1**	9.2–13.7	6.0–10.4	5.8–9.4	61–122	62–111	53–102	5–18	1–6	1–5
**h^2^ = 0.8, E = 0.1**	11.2–13.0	5.6–10.6	5.1–9.5	69–118	54–77	44–94	6–20	2–8	1–5
**h^2^ = 0.1, E = 0.5**	10.5–14.3	5.7–9.8	5.1–7.8	75–120	57–86	48–103	5–18	2–7	1–2
**h^2^ = 0.2, E = 0.5**	12.0–18.7	6.4–10.2	5.7–7.0	131–137	54–95	37–71	3–17	2–7	1–4
**h^2^ = 0.4, E = 0.5**	12.1–18.5	5.5–11.7	5.0–7.2	122–129	83–99	41–74	5–18	3–7	1–5
**h^2^ = 0.8, E = 0.5**	11.2–18.3	5.8–10.5	5.1–8.6	76–126	52–107	45–96	6–21	2–9	2–5

Values reflect the lows and highs obtained using various marker subsets (from 500 markers to all markers). Note that larger cosine values are equivalent to smaller p-values.

### Results with Field Performance Data

In addition to the simulations, the predictive ability of each method was evaluated using maize and barley data reported by CIMMYT [24] and BarleyCAP, respectively. With the maize dataset, five-fold CV was implemented with the traits ASI and GY to evaluate predictive ability and compare methods. For maize ASI, the RKHS-M method produced the highest correlation of 0.554, followed closely by pRKHS-NE and pRKHS-E (Table 5). For maize GY, the RHKS-M method produced the highest correlation of 0.447, followed closely by pRHKS-NE, RR-BLUP, pRHKS-E, and BayesB. The pRKHS method outperformed RR-BLUP, BayesA, and BayesB with ASI and had comparable performance in GY with RR-BLUP and BayesB, with pRKHS-E and pRKHS-NE performing nearly identically. With regard to pRKHS-E and pRKHS-NE, the optimal numbers of markers contributing to phenotypic variation were 700 and 600, and SPCs explaining 70% and 65% of data matrix variance, respectively, were included in the model in order to achieve high prediction accuracy for ASI trait. More markers were involved in GY, *i.e*. 1000 and 900 markers for pRKHS-E and pRKHS-NE, respectively, suggesting that more genes and perhaps more epistasis was involved in trait expression (Table 4). Furthermore, the optimum CoV for pRKHS-E was 0.3 for both ASI and GY.

**Table 5:** Applying pRKHS to real life scenarios, Pearson correlation coefficients between estimated breeding value (EBV) and phenotype obtained from five-fold cross-validation (CV) implemented for maize anthesis-silking interval (ASI) and grain yield (GY) for each of the 6 statistical methods.

Trait	CV	Methods	MarkerNumber	%PC	Correlation
ASI	CV	RR-BLUP			0.495
	CV	BayesA			0.388
	CV	BayesB			0.495
	CV	RKHS-M			0.554
	CV	pRKHS-E	700	70%	0.520
	CV	pRKHS-NE	600	65%	0.526
GY	CV	RR-BLUP			0.423
	CV	BayesA			0.392
	CV	BayesB			0.421
	CV	RKHS-M			0.447
	CV	pRKHS-E	1000	75%	0.422
	CV	pRKHS-NE	900	65%	0.425

The optimal number of markers contributing to phenotypic variation and percent of variations explained by the included SPCs were shown for pRKHS methods; results were averaged across five repeated fittings. Optimal cosine value was 0.3 for pRKHS-E across all datasets.

A three-year set of experimental data from BarleyCAP was used to measure the predictive ability given an independent set of breeding lines (Table 6). Phenotypes (*i.e.* grain yield (GYD) and plant height (PHT)) and genotypes from Year 2007 were used to fit models and evaluate ten-fold CV performance. The fitted models were then used to predict the phenotype of a different set of 96 lines in Year 2008 and 57 lines in Year 2009. For GYD, the pRKHS method substantially outperformed other methods for predicting 2008 and 2009 phenotypes, with pRKHS-E performing better with 2008 predictions and pRKHS-NE performing better with 2009 predictions. The optimal numbers of markers contributing to phenotypic variation were 1500 and 800 and SPCs explaining 70% and 75% of marker variation were included in the model in order to attain high correlation for pRKHS-E and pRKHS-NE, respectively. For PHT, the optimal number of markers was 1000 and SPCs explaining 75% of the variation were included in both pRKHS-E and pRKHS-NE methods. pRKHS-NE generated the highest correlation in 2007 and 2009 data sets whereas pRKHS-E had the highest correlation of EBV and 2008 PHE. Optimal CoV for pRKHS-E was found at 0.3 in both traits.

**Table 6:** Applying pRKHS to real life scenarios, Pearson correlation coefficients between estimated breeding value (EBV) and phenotype obtained from ten-fold CV using genotypes and phenotypes of barley lines in year 2007 and prediction based on genotypes of different lines in year 2008 and 2009 implemented for grain yield (GYD) and plant height (PHT) for each of the 6 statistical methods.

Traits	Year	Methods	MarkerNumber	%PC	Correlation
GYD	2007	RR-BLUP			0.449
	2007	BayesA			0.448
	2007	BayesB			0.510
	2007	RKHS-M			0.260
	2007	pRKHS-E	1500	70%	0.438
	2007	pRKHS-NE	800	75%	0.538
	2008	RR-BLUP			0.104
	2008	BayesA			0.073
	2008	BayesB			0.108
	2008	RKHS-M			-0.009
	2008	pRKHS-E	1500	70%	0.295
	2008	pRKHS-NE	800	75%	0.188
	2009	RR-BLUP			0.052
	2009	BayesA			0.085
	2009	BayesB			0.047
	2009	RKHS-M			0.130
	2009	pRKHS-E	1500	70%	-0.081
	2009	pRKHS-NE	800	75%	0.148
PHT	2007	RR-BLUP			0.447
	2007	BayesA			0.446
	2007	BayesB			0.460
	2007	RKHS-M			0.514
	2007	pRKHS-E	1000	75%	0.465
	2007	pRKHS-NE	1000	75%	0.520
	2008	RR-BLUP			-0.015
	2008	BayesA			-0.006
	2008	BayesB			-0.049
	2008	RKHS-M			-0.083
	2008	pRKHS-E	1000	75%	0.084
	2008	pRKHS-NE	1000	75%	0.062
	2009	RR-BLUP			0.076
	2009	BayesA			0.111
	2009	BayesB			0.107
	2009	RKHS-M			0.191
	2009	pRKHS-E	1000	75%	0.203
	2009	pRKHS-NE	1000	75%	0.222

The optimal number of markers contributing to phenotypic variation and percent of variations explained by the included SPCs were shown for pRKHS methods; results were averaged across five repeated fittings. Optimal cosine value was 0.3 for pRKHS-E across all datasets.

## Discussion

This study demonstrates the advantages of using nonparametric methods to estimate breeding value and to predict phenotypic performance, especially for traits involving epistatic gene action. The new method is novel because it features a new combination of supervised principal component analysis and reproducing kernel Hilbert spaces, both established statistical methods. The introduction of SPCA complements RKHS by reducing dimensionality and background noise. Two versions of the method were devised to span the range of epistasis involved in trait expression, with pRKHS-E designed to account for low/moderate to high epistasis and pRKHS-NE accommodating circumstances in which no/minimal epistasis exists in the target trait. To evaluate the performance of the pRKHS method, three other shrinkage methods and another nonparametric method RKHS-M were compared. The results obtained from simulation confirmed that in the absence of epistasis, pRKHS-NE performs comparably with BayesB and better than pRKHS-E (Table 1). When epistasis is present, pRKHS-E shows better predictive ability among all other methods (Tables 2, 3). In addition, results with actual data show that the pRKHS method consistently outperforms shrinkage methods and performed comparably to RKHS-M, further confirming the predictive ability of the pRKHS method in real application.

According to selection theory, MBS holds advantage over PS when the genetic correlation (correlation between estimated breeding value and true breeding value) is higher than the correlation of mid-parent and offspring. Given that pRKHS outperformed PS in most of the cases (Tables 1, 2, 3), its potential use to facilitate indirect selection based on marker information alone is highlighted. However, pRKHS-E and pRKHS-NE are not expected to perform equivalently due to different statistical models on which they are based. In the absence of epistasis, overall underperformance of pRKHS-E (Table 1) is mostly attributed to model overfitting. This may be further supported by the results that pRKHS-E had high r_EBV:PHE_ but also the lowest r_EBV:TBV_ among six methods at h^2^ = 0.8 (Table 1), which suggests estimates from pRKHS-E have higher variance and are more biased in the absence of epistasis. As epistasis was increased in simulation scenarios, r_EBV:TBV_ of pRKHS-E decreased slowly (Table 2, Table 3), suggesting properly modeling epistasis upholds the advantages of applying MBS.

Note that correlations with pRKHS-E are not overwhelmingly higher compared to pRKHS-NE in low epistasis scenarios (Table 2). The result that pRKHS-E outperforms pRKHS-NE in only five out of eight cases indicates pRKHS-NE may function well when a low level of epistasis impacts trait expression. The above phenomenon may be explained by the fact that the optimal CoV for pRKHS-E was 0.3 in low epistasis scenario, wherein about two to three SPC interactions on average were involved in the model (Table 4). Overall, 18 SPCs were needed to explain around 70% of the variation and these were included as main effects in the pRKHS-E and pRKHS-NE models (Figure 1). Since the principal component score is a linear combination of the weighted marker score, the linear combination of 18 SPC scores may account for a few of the SPC interactions. The above argument is further supported by the observations that fitting model using CoV of 0.2 (*i.e.* more SPC interactions) causes multicollinearity in some cases. Overall, features of principal component scores may help the additive model pRKHS-NE fit well in the situation of low epistatic interactions.

Cosine threshold value as mentioned in this study is a nonparametric model diagnostic and used as a criterion to select SPC interactions. As the counterpart of F-statistics in a parametric model [17], CoV could theoretically be transformed to a test statistic similar to the F-distribution p-values with some modification [35]. However, the degrees of freedom for F-distribution which are estimated from the trace of the smoothing matrix change every time a new pair of SPC interactions is fitted. Therefore, the consequential p-value is not monotone with the cosine value, indicating the same cosine value could be assigned for different p-values in different model fitting, which is misleading to SPC interaction selection, causing loss or false inclusion of interactions. We did some preliminary experimentation by constructing models using a transformed p-value instead of direct CoV for SPC interaction selection and found low predictive ability (data not shown).

In addition to predictive performance, comparisons between pRKHS and other methods can consider computational load. Several studies [8], [36] have suggested the computational advantages of using nonparametric methods over shrinkage methods. For our models, the cost of the RKHS algorithm is 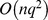, where *n* is sample size and *q* is number of dimensions. With SPCA, *q* is usually around 18 to 20 (Figure1), indicating computational time of pRKHS will be mainly impacted by sample size instead of marker number. With pRKHS, most of the computational load involves constructing reproducing kernels and the smoothing matrix and estimating smoothing parameter *λ*. In contrast, the computation load with Bayesian shrinkage methods is linearly related to the number of features since these are Markov Chain Monte Carlo (MCMC) based, with computational time increasing as the number of number of markers increases.

Model performances were influenced by the underlying genetic architecture of the trait of interest. pRKHS plays an important role when trait expression is influenced by epistasis, whereas shrinkage methods may have higher predictive ability when a trait is controlled by strictly additive gene effects. The genetic architecture represented by BayesB assumes a trait is controlled by a few genes with large effects and many genes with small effects. BayesB further allows some of the markers to have zero effect, suggesting a nonuniform distribution of genes contributing to phenotypic variation throughout the genome [2]. Thus, among the three shrinkage methods in this evaluation, BayesB has the most in common with pRKHS with respect to the genetic simulation. Fair approximation of the underlying genome seems to contribute to the good performance of BayesB and pRKHS.

In general, RKHS methods performed better than shrinkage methods. Comparing nonparametric methods, pRKHS performs better than RKHS-M in both simulation and the barley data scenarios (Tables 1, 2, 3, 6) but lower in maize data scenarios (Table 5). These differences in performance might be attributed to two factors: 1) model specifications such as tuning parameter and reproducing kernel, and 2) differences in genetic architecture. The smoothing parameters 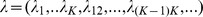 place different weights to different main effects and interactions, *i.e.* downplays the effect of unimportant predictors and provides better predictions. The smoothing parameters *λ* in pRKHS were tuned using the data-driven “GCV” score during model training, while the bandwidth parameter *φ* in RKHS-M was set to sample median of the squared Euclidean distance as mentioned by Crossa *et al.* [24]. Meanwhile, we used polynomial kernels to construct kernel matrix while Gaussian kernel was adopted by RKHS-M. To measure the impact of using only one kernel in RKHS-M, kernel averaging model, *i.e*. K_2_+K_7_ developed in de los Campos *et al.* [36] was also applied on barley data and similar results were obtained (data not shown). It is worth noting that Gianola and van Kaam [8] included parametric mixed effects besides nonparametric function. Such an extension has been also built in the *ssanova* function [37]. In short, both RKHS-M and pRKHS have their own advantages of prediction, *i.e.* RKHS-M had higher predictive ability with maize data (Table 5) while pRKHS excel with barley data (Table 6). A combined usage of RKHS-M and pRKHS may outperform the usage of a single method.

The low prediction accuracy observed in “2008” and “2009” as near zero correlation values in Table 6 may be attributed to several reasons. In particular, the three-year barley data are pedigree-independent of each other, suggesting the training and testing datasets are mostly independent. The scenario is different from cross validation within one year (*i.e*. “2007” correlation values in Table 6), in which case the barley lines are pedigree-related to a certain degree; thus, the training and testing data within a year have more relationship than those across years, leading to higher prediction accuracy.

The predictive ability of pRKHS is highly related to the included SPCs and their interactions (for pRKHS-E). Bair *et al.* [23] suggested use of the first several SPCs for prediction and later Li *et al.* [38] applied the first three SPCs in genome-wide association mapping. With our methods, the number of SPCs to include is flexible and depends on the extent of epistasis; it is quantified by selecting the proportion of variation instead of specific numbers. Empirically, we found that with setting a threshold of around 70% as the amount of the variation explained by the model and then utilizing only the SPCs associated with that threshold, an appropriate balance between variance explained and goodness-of-fit was achieved in most of the cases through simulation, and this was confirmed with real data applications (Table 5). However, depending on the crop data and the genetic architecture, the optimal threshold may actually vary by ±10%, indicating a range of 60% to 80% to achieve best predictability. Cross-validation could be used to find the best number of SPCs to include for a specific data.

Optimal marker density for prediction is a topic of great debate. Some studies advocate use of all markers with dense coverage [2], while others found little value in dense coverage of the genome and advocate use of a reduced set of markers for prediction [22], [39], [40]. Ways of selecting markers also vary and can be based on random selection, genetic distance or LD extent, or entropy reduction, for example. In this study, selection of makers was based on the magnitude of the regression coefficient, *i.e.* the size of the marker effect, and the prediction accuracy is actually increased by discarding certain markers which contribute little to the target trait.

The reduced marker approach used with the new pRKHS method seems to confer some advantages. When no epistasis is present, Bayesian methods perform well with utilization of the full marker information. However, the results that pRKHS-NE had slightly lower prediction accuracy than BayesB (Table 1) suggest a near-similar level of predictive ability may be enabled even if partial marker information is used. With pRKHS methods, a ‘preselection’ procedure is applied before doing PCA to filter out “non-significant” markers. This increases the probability that the subsequent supervised principal components are in good association with the trait of interest [23]. More importantly, the nonlinearity feature of SPCA which is due to initial marker selection falls into the category of RKHS regression well. Furthermore, PCA serves not only for dimension reduction but also clustering. In simulation, influential markers of each SPC except the first SPC, which contains markers from all ten chromosomes, usually come from one or two linkage groups (chromosomes). Therefore, one SPC is considered to be one or two large haplotypes and the SPC interaction presents the haplotype interactions instead of single marker interaction. It is tedious to evaluate the interaction effect between every pair of SNP markers using a dense marker set; however, pRKHS allows us to narrow down the potential SNP interaction effects by investigating the influential markers of two SPCs which have significant interaction effects with each other. Furthermore, methods using haplotypes have been proved to show higher predictive ability than those only using single marker [41], [42].

Besides prediction, use of pRKHS facilitates inferences about the extent of epistasis involved with a trait of interest. For maize trait ASI with heritability estimated at 0.8 [43], pRKHS-E with optimal cosine value of 0.3 and pRKHS-NE produced comparable results and outperformed RR-BLUP, BayesA, and BayesB that only include additive effects (Table 5), indicating that inclusion of a few pairs of SPC interactions in the model increases prediction. The above results not only correspond to the case of simulated low epistasis scenario with h^2^ = 0.8 (Table 2) but also are consistent with the conclusions by Buckler *et al.* [43] who suggested that ASI may involve some low level of epistasis. For GYD and PHT in barley, Xu and Jia [44] concluded that epistasis contributes little to genetic variance for self-pollinated species based on work with a doubled haploid population derived from cultivated parents, although Von Korff *et al.* [45] later found strong epistatic interactions existed in plant height and yield traits in barley and attributed the reason to use of exotic parents and different statistical approaches. As shown in Table 6, our results align with the low epistasis conclusions from Xu and Jia [44] as pRKHS-E involving a few interactions (cosine value equals 0.3) and pRKHS-NE have comparable predictive ability and both methods are more predictive than others.

Overall, the pRKHS method performs well in estimating breeding value and predicting performance when epistasis explains certain proportion of the phenotypic variation. The rate of genetic gain may be enhanced to a certain degree depending on the underlying epistatic extent. Furthermore, pRKHS can be adapted to different types of genetic architectures, *i.e.* epistatic extent and linkage disequilibrium, through tuning CoV(representing epistatic strength) and MS (representing the proportion of markers contributing to the target trait), respectively. In cases where no prior knowledge of genetic architecture are known, running methods pertaining to different genetic architectures, such as RR-BLUP (infinitesimal model), BayesB (finite loci model) and pRKHS methods (epistasis model) are recommended. Compared to other methods, pRKHS is not only for prediction purposes but also has the capacity to facilitate inferences about the extent of epistasis involved with a trait of interest, which helps scientists to unravel mysteries about the genetic architecture of complex traits. The new nonparametric methods can be readily extended to account for dominance effects and other semi-parametric methods of dealing with some covariates, *e.g.* population structure, typically managed in a parametric manner.

## Supporting Information

Appendix S1 **Further detail related to the nonparametric model for pRKHS.**
(DOCX)

R Code S1 **R code provided to reproduce pRKHS statistics.**
(PDF)

## References

[1] Fernando R, Grossman M (1989) Marker assisted selection using best linear unbiased prediction. Genet Sel Evol 21: 467–477.

[2] Meuwissen THE, Hayes BJ, Goddard ME (2001) Prediction of total genetic value using genome-wide dense marker maps. Genetics 157: 1819–1829. 11290733 10.1093/genetics/157.4.1819 PMC1461589

[3] Gruber MHJ (1998) Improving efficiency by shrinkage. New York: Marcel Dekker.

[4] Xu S (2003) Estimating polygenic effects using markers of the entire genome. Genetics 163: 789–801. 12618414 10.1093/genetics/163.2.789 PMC1462468

[5] de los Campos G, Naya H, Gianola D, Crossa J, Legarra A, et al (2009) Predicting quantitative traits with regression models for dense molecular markers and pedigrees. Genetics 182: 375–385. 19293140 10.1534/genetics.109.101501 PMC2674834

[6] Dudley JW, Johnson GR (2009) Epistatic models improve prediction of performance in corn. Crop Sci 49: 1533–1533.

[7] Cockerham CC (1954) An extension of the concept of partitioning hereditary variacne for analysis of covariances among relatives when epistasis is present. Genetics 39: 859–882. 17247525 10.1093/genetics/39.6.859 PMC1209694

[8] Gianola D, van Kaam JBCHM (2008) Reproducing kernel hilbert spaces regression methods for genomic assisted prediction of quantitative traits. Genetics 178: 2289–2303. 18430950 10.1534/genetics.107.084285 PMC2323816

[9] Gonzalez-Recio O, Gianola D, Long N, Weigel KA, Rosa GJM, et al (2008) Nonparametric methods for incorporating genomic information into genetic evaluations: an application to mortality in broilers. Genetics 178: 2305–2313. 18430951 10.1534/genetics.107.084293 PMC2323817

[10] Gianola D, Fernando RL, Stella A (2006) Genomic-assisted prediction of genetic value with semiparametric procedures. Genetics 173: 1761–1776. 16648593 10.1534/genetics.105.049510 PMC1526664

[11] Mallick BK, Ghosh D, Ghosh M (2005) Bayesian classification of tumours by using gene expression data. J Roy Stat Soc Ser B (Statistical Methodology) 67: 219–234.

[12] Bennewitz J, Solberg T, Meuwissen T (2009) Genomic breeding value estimation using nonparametric additive regression models. Genet Sel Evol 41: 20. 19284696 10.1186/1297-9686-41-20 PMC2657215

[13] Nadaraya E (1964) On estimating regression. Theor Probab Appl 9: 141–142.

[14] Watson G (1964) Smooth regression analysis. Sankhya A 26: 359–372.

[15] Fan J (1996) Local polynomial modelling and its applications. Boca Raton, Florida: Chapman & Hall/CRC.

[16] Wahba G (1990) Spline models for observational data: SIAM, Philadelphia.

[17] Gu C (2002) Smoothing spline ANOVA models. New York: Springer-Verlag.

[18] Wang Y (2011) Smoothing Splines: Methods and Applications: Chapman & Hall/CRC.

[19] Macciotta NPP, Gaspa G, Steri R, Nicolazzi EL, Dimauro C, et al (2010) Using eigenvalues as variance priors in the prediction of genomic breeding values by principal component analysis. J Dairy Sci 93: 2765–2774. 20494186 10.3168/jds.2009-3029

[20] Macciotta N, Gaspa G, Steri R, Pieramati C, Carnier P, et al (2009) Pre-selection of most significant SNPS for the estimation of genomic breeding values. BMC Proc 3: S14. 10.1186/1753-6561-3-s1-s14 PMC2654495 19278540

[21] Schulz-Streeck T, Ogutu J, Piepho H-P (2011) Pre-selection of markers for genomic selection. BMC Proc 5: S12. 10.1186/1753-6561-5-S3-S12 PMC3103197 21624168

[22] Long N, Gianola D, Rosa GJM, Weigel KA, Avendaño S (2007) Machine learning classification procedure for selecting SNPs in genomic selection: application to early mortality in broilers. J Anim Breed Genet 124: 377–389. 18076475 10.1111/j.1439-0388.2007.00694.x

[23] Bair E, Hastie T, Paul D, Tibshirani R (2006) Prediction by supervised principal components. J Am Stat Assoc 101: 119–137.

[24] Crossa J, de los Campos G, Perez P, Gianola D, Burgueno J, et al (2010) Prediction of genetic values of quantitative traits in plant breeding using pedigree and molecular markers. Genetics 186: 713–724. 20813882 10.1534/genetics.110.118521 PMC2954475

[25] Bernardo R, Yu J (2007) Prospects for genomewide selection for quantitative traits in maize. Crop Sci 47: 1082–1090.

[26] Fu Y, Wen TJ, Ronin YI, Chen HD, Guo L, et al (2006) Genetic dissection of intermated recombinant inbred lines using a new genetic map of maize. Genetics 174: 1671–1683. 16951074 10.1534/genetics.106.060376 PMC1667089

[27] Kosambi DD (1944) The estimation of map distance from recombination values. Annals of Eugenics 12: 172–175.

[28] Zeng ZB, Wang T, Zou W (2005) Modeling quantitative trait loci and interpretation of models. Genetics 169: 1711–1725. 15654105 10.1534/genetics.104.035857 PMC1449562

[29] Lande R, Thompson R (1990) Efficiency of marker-assisted selection in the improvement of quantitative traits. Genetics 124: 743–756. 1968875 10.1093/genetics/124.3.743 PMC1203965

[30] Hastie T, Tibshirani R, Friedman J (2009) The Elements of Statistical Learning 2nd ed.: Springer.

[31] Habier D, Fernando R, Kizilkaya K, Garrick D (2011) Extension of the bayesian alphabet for genomic selection. BMC Bioinformatics 12: 186. 21605355 10.1186/1471-2105-12-186 PMC3144464

[32] Gu C (2011) gss: General Smoothing Splines. R package version 1.1–7. Available: http://CRAN.R-project.org/package=gss. Accessed 2012 Oct 30.

[33] Plummer M, Best N, Cowles K, Vines K (2006) coda: output analysis and diagnostics for MCMC. R News 6: 7–11.

[34] Falconer DS, Mackay TFC (1996) Introduction to quantitative genetics. Essex, UK: Longman and Company.

[35] Ma P, Zhong WX, Liu JS (2009) Identifying differentially expressed genes in time course microarray data. Stat in Biosciences 1: 144–159.

[36] de los Campos G, Gianola D, Rosa JMG, Weigel AK, Crossa J (2010) Semi-parametric genomic-enabled prediction of genetic values using reproducing kernel Hilbert spaces methods. Genet Res 92: 295–308. 10.1017/S0016672310000285 20943010

[37] Gu C, Ma P (2005) Optimal smoothing in nonparametric mixed-effect models. Annals of Statistics 33: 1357–1379.

[38] Li J, Das K, Fu G, Li R, Wu R (2011) The Bayesian lasso for genome-wide association studies. Bioinformatics 27: 516–523. 21156729 10.1093/bioinformatics/btq688 PMC3105480

[39] Luan T, Woolliams JA, Lien S, Kent M, Svendsen M, et al (2009) The accuracy of genomic selection in norwegian red cattle assessed by cross validation. Genetics 183: 1119–1126. 19704013 10.1534/genetics.109.107391 PMC2778964

[40] VanRaden P, Van Tassell C, Wiggans G, Sonstegard T, Schnabel R, et al (2009) Reliability of genomic predictions for North American Holstein bulls. J Dairy Sci 92: 16–24. 19109259 10.3168/jds.2008-1514

[41] Akey J, Jin L, Xiong M (2001) Haplotypes vs single marker linkage disequilibrium tests: what do we gain? Eur J Hum Genet 9: 291–300. 11313774 10.1038/sj.ejhg.5200619

[42] Calus MPL, Meuwissen THE, de Roos APW, Veerkamp RF (2008) Accuracy of genomic selection using different methods to define haplotypes. Genetics 178: 553–561. 18202394 10.1534/genetics.107.080838 PMC2206101

[43] Buckler ES, Holland JB, Bradbury PJ, Acharya CB, Brown PJ, et al (2009) The genetic architecture of maize flowering time. Science 325: 714–718. 19661422 10.1126/science.1174276

[44] Xu S, Jia Z (2007) Genomewide analysis of epistatic effects for quantitative traits in barley. Genetics 175: 1955–1963. 17277367 10.1534/genetics.106.066571 PMC1855123

[45] Von Korff M, Léon J, Pillen K (2010) Detection of epistatic interactions between exotic alleles introgressed from wild barley (*H. vulgare* ssp. *spontaneum*). Theor Appl Genet 121: 1455–1464. 20617300 10.1007/s00122-010-1401-y

