# Supplementary material for: Nonparametric Method for Genomics-Based Prediction of Performance of Quantitative Traits Involving Epistasis in Plant Breeding
Source: PLoS One. 2012 Nov 30;7(11):e50604. doi: 10.1371/journal.pone.0050604 (PMC3511520; doi:10.1371/journal.pone.0050604)
Supplement: Appendix S1 — Further detail related to the nonparametric model for pRKHS. (DOCX) [file pone.0050604.s001.docx]

**Appendix**

We modeled the phenotype and SPCs using a general nonparametric model on domain  = [0, 1], which can be written as

$Y_{i}=\eta(\mathbf{x}_{i})+\varepsilon_{i}$,

where Y_i_ is the phenotype of *i*th individual, $\mathbf{x}_{i}=\left(x_{i}^{(1)},x_{i}^{(2)}...,x_{i}^{(K)}\right)$ is the vector of *k* SPCs $x_{i}^{(1)},x_{i}^{(2)}...,x_{i}^{(K)}$of *i*th individual, $\eta$ is some unknown K-variate function relating SPCs and phenotype, and $\varepsilon_{i}\sim N\left(0,\sigma^{2}\right)$ is error term for *i*th individual. Analogous to classical ANOVA in linear models, a functional ANOVA decomposition could be written as,

$\eta(\mathbf{x})=\eta_{0}+\sum_{j=1}^{K} \eta_{j}\left(x^{(j)}\right)+\sum_{j=1}^{K} \sum_{m=j+1}^{K} \eta_{jm}\left(x^{(j)},x^{(m)}\right)+\text{all higher order of interactions}$,

where $\eta_{0}$ is a constant representing baseline effect,$\eta_{j}\text{'s}$ are the main effects, and $\eta_{jm}\text{'s}$ are the two-way interactions. Each $\eta_{j}$ was estimated in a RKHS , and each $\eta_{jm}$ was estimated in the tensor product RKHS , and so on [[17](#_ENREF_17)]. $\eta(\mathbf{x})$was thus estimated by a penalized least squares in the RKHS,

, (1)

where , $\lambda_{k}$’s are the inter-space (rescaling) smoothing parameters, $P_{k}$’s are orthogonal projection of $\eta$to RKHS and *λ* denotes all$\lambda_{k}$’s . The first term $\sum_{i=1}^{n} \left(Y_{i}-\eta(\mathbf{x}_{i})\right)^{2}$ measures the goodness-of-fit, the second term penalizes for smoothness of the *η*, and the smoothing parameter $\lambda$ strikes the balance between the goodness-of-fit and smoothness of the *η*. The minimizer of (1) has expression

, (2)

whereis the basis of null spaceand coefficients$d_{v}$and$c_{j}$need to be estimated from data. The (2) could be expressed in a matrix form:

$\left(\mathbf{Y}-S\mathbf{d}-Q\mathbf{c}\right)^{T}\left(Y-S\mathbf{d}-Q\mathbf{c}\right)+n\lambda\mathbf{c}^{T}Q\mathbf{c}$, (3)

where *S* is *n* x *m* matrix with its $\left(i,v\right)th$ entry to be and *Q* is *n* x *n* kernel matrix with the $\left(i,j\right)th$ entry to be and c and d are unknown coefficients. Normal equations of (3) are:

$\begin{matrix} Q\left(Q+n\lambda I\right)\mathbf{c}+QS\mathbf{d}=Q\mathbf{Y} \\ S^{T}Q\mathbf{c}+S^{T}S\mathbf{d}=S^{T}\mathbf{Y} \end{matrix}$.

Assuming *S* has full column rank, we can solve the normal equation in a numerically stable approach through the QR-decomposition [[17](#_ENREF_17)]. And the fitted value is

$\begin{matrix} \hat{\mathbf{Y}}=\left(\eta_{\lambda}\left(\mathbf{x}_{1}\right),...,\eta_{\lambda}\left(\mathbf{x}_{n}\right)\right)^{T} \\ \text{ }=S\hat{\mathbf{d}}+Q\hat{\mathbf{c}} \\ \text{ }=\left(I-n\lambda\left(M^{-1}-M^{-1}S(S^{T}M^{-1}S)^{-1}S^{T}M^{-1}\right)\right)\mathbf{Y} \\ \text{ = }A\left(\lambda\right)\mathbf{Y}\text{ }, \end{matrix}$ (4)

where $M=Q+n\lambda I$, and $A\left(\lambda\right)=I-n\lambda\left(M^{-1}-M^{-1}S(S^{T}M^{-1}S)^{-1}S^{T}M^{-1}\right)$ is the counterpart of projection matrix in parametric model, called smoothing matrix. Smoothing parameter was selected by using the generalized cross validation (GCV) score [[17](#_ENREF_17)]:

. (5)

In the pRKHS-NE version, we consider, and in the pRKHS-E version, we consider. When endowed with a certain inner product, we obtain K+1 ,i.e, $1,k_{1}(x_{i}^{(1)}),k_{1}(x_{i}^{(2)}),\ldots,k_{1}(x_{i}^{(K)})$ , and

where$k_{1}\left(t\right)=t-0.5$,, andfor pRKHS-NE.

For pRKHS-E, we have K(K+1)/2+1 .i.e., $1,k_{1}(x_{i}^{(1)}),k_{1}(x_{i}^{(2)}),\ldots,k_{1}(x_{i}^{(K)}),k_{1}(x_{i}^{(1)})k_{1}(x_{i}^{(2)}),\ldots,k_{1}(x_{i}^{(K-1)})k_{1}(x_{i}^{(K)}),$
