# Supplementary material for: Nonparametric Method for Genomics-Based Prediction of Performance of Quantitative Traits Involving Epistasis in Plant Breeding
Source: PLoS One. 2012 Nov 30;7(11):e50604. doi: 10.1371/journal.pone.0050604 (PMC3511520; doi:10.1371/journal.pone.0050604)
Supplement: R Code S1 — R code provided to reproduce pRKHS statistics. (PDF) [file pone.0050604.s002.pdf]

```
Red.RKHS <- function(y_train,x_train,y_test,x_test,maknum,PV,cosine,P){
  require(gss)
  mkterm <- function(mf, type){
    mt <- attr(mf, "terms")
    xvars <- as.character(attr(mt, "variables"))[-1]
    xfacs <- attr(mt, "factors")
    term.labels <- labels(mt)
    if (attr(attr(mf, "terms"), "intercept"))
        term.labels <- c("1", term.labels)
    vlist <- xvars[as.logical(apply(xfacs, 1, sum))]
    if (!is.null(type) && !is.list(type) && (type %in% c("cubic",
        "linear", "tp"))) {
        type.wk <- type
        type <- NULL
        for (xlab in vlist) type[[xlab]] <- type.wk
    }
    var.type <- NULL
    for (xlab in vlist) {
        x <- mf[, xlab]
        if (!is.null(type[[xlab]])) {
            type.wk <- type[[xlab]][[1]]
            if (!(type.wk %in% c("ordinal", "nominal", "cubic",
                "linear", "per", "cubic.per", "linear.per", "tp",
                "sphere", "custom")))
                stop("gss error in mkterm: unknown type")
            if (type.wk %in% c("ordinal", "nominal")) {
                par.wk <- NULL
                if (!is.factor(x))
                  stop("gss error in mkterm: wrong type")
            }
            if (type.wk %in% c("cubic", "linear")) {
                if (length(type[[xlab]]) == 1) {
                  mn <- min(x)
                  mx <- max(x)
                  par.wk <- c(mn, mx) + c(-1, 1) * 1 * (mx -
                    mn)
                }
                else par.wk <- type[[xlab]][[2]]
                if (is.factor(x) | !is.vector(x))
                  stop("gss error in mkterm: wrong type")
            }
            if (type.wk %in% c("per", "cubic.per", "linear.per")) {
                if (type.wk == "per")
                  type.wk <- "cubic.per"
                if (length(type[[xlab]]) == 1)
                  stop("gss error in mkterm: missing domain of periodicity")
                else par.wk <- type[[xlab]][[2]]
                if (is.factor(x) | !is.vector(x))
                  stop("gss error in mkterm: wrong type")
            }
            if (type.wk == "tp") {
                if (length(type[[xlab]]) == 1)
                  par.wk <- list(order = 2, mesh = x, weight = 1)
                else {
                  par.wk <- par.wk1 <- type[[xlab]][[2]]
                  if (length(par.wk1) == 1)
                    par.wk <- list(order = par.wk1, mesh = x,
                      weight = 1)
                  if (is.null(par.wk$mesh))
                    par.wk$mesh <- x
                  if (is.null(par.wk$weight))
                    par.wk$weight <- 1
                }
                if (dim(as.matrix(x))[2] != dim(as.matrix(par.wk$mesh))[2])
                  stop("gss error in mkterm: wrong dimension in normalizing mesh")
            }
            if (type.wk == "sphere") {
```

```
                if (length(type[[xlab]]) == 1)
                  par.wk <- 2
                else par.wk <- type[[xlab]][[2]]
                if (!(par.wk %in% (2:4)))
                  stop("gss error in mkterm: spherical order not implemented")
            }
            if (type.wk == "custom")
                par.wk <- type[[xlab]][[2]]
        }
        else {
            if (is.factor(x)) {
                if (is.ordered(x))
                  type.wk <- "ordinal"
                else type.wk <- "nominal"
                par.wk <- NULL
            }
            else {
                if (is.vector(x)) {
                  type.wk <- "cubic"
                  mn <- min(x)
                  mx <- max(x)
                  par.wk <- c(mn, mx) + c(-1, 1) * 1 * (mx -
                    mn)
                }
                else {
                  type.wk <- "tp"
                  par.wk <- list(order = 2, mesh = x, weight = 1)
                }
            }
        }
        var.type[[xlab]] <- list(type.wk, par.wk)
    }
    term <- list(labels = term.labels)
    iphi.wk <- 1
    irk.wk <- 1
    for (label in term.labels) {
        iphi <- irk <- phi <- rk <- NULL
        if (label == "1") {
            iphi <- iphi.wk
            iphi.wk <- iphi.wk + 1
            term[[label]] <- list(iphi = iphi, nphi = 1, nrk = 0)
            next
        }
        vlist <- xvars[as.logical(xfacs[, label])]
        x <- mf[, vlist]
        dm <- length(vlist)
        if (dm == 1) {
            type.wk <- var.type[[vlist]][[1]]
            if (type.wk %in% c("nominal", "ordinal")) {
                if (type.wk == "nominal")
                  fun.env <- mkrk.nominal(levels(x))
                else fun.env <- mkrk.ordinal(levels(x))
                if (nlevels(x) > 2) {
                  nphi <- 0
                  rk.fun <- function(x, y, nu = 1, env, outer.prod = FALSE) {
                    env$fun(x, y, env$env, outer.prod)
                  }
                  nrk <- 1
                  irk <- irk.wk
                  irk.wk <- irk.wk + nrk
                  rk <- list(fun = rk.fun, env = fun.env)
                }
                else {
                  phi.fun <- function(x, nu = 1, env) {
                    wk <- as.factor(names(env$env$code)[1])
                    env$fun(x, wk, env$env)
                  }
```

```
                  nphi <- 1
                  iphi <- iphi.wk
                  iphi.wk <- iphi.wk + nphi
                  phi <- list(fun = phi.fun, env = fun.env)
                  nrk <- 0
                }
            }
            if (type.wk == "cubic") {
                range <- var.type[[vlist]][[2]]
                phi.env <- mkphi.cubic(range)
                phi.fun <- function(x, nu = 1, env) env$fun(x,
                  nu, env$env)
                nphi <- 1
                iphi <- iphi.wk
                iphi.wk <- iphi.wk + nphi
                phi <- list(fun = phi.fun, env = phi.env)
                rk.env <- mkrk.cubic(range)
                rk.fun <- function(x, y, nu = 1, env, outer.prod = FALSE) {
                  env$fun(x, y, env$env, outer.prod)
                }
                nrk <- 1
                irk <- irk.wk
                irk.wk <- irk.wk + nrk
                rk <- list(fun = rk.fun, env = rk.env)
            }
            if (type.wk %in% c("cubic.per", "linear", "linear.per",
                "sphere")) {
                range <- var.type[[vlist]][[2]]
                nphi <- 0
                if (type.wk == "cubic.per")
                  rk.env <- mkrk.cubic.per(range)
                if (type.wk == "linear")
                  rk.env <- mkrk.linear(range)
                if (type.wk == "linear.per")
                  rk.env <- mkrk.linear.per(range)
                if (type.wk == "sphere")
                  rk.env <- mkrk.sphere(range)
                rk.fun <- function(x, y, nu = 1, env, outer.prod = FALSE) {
                  env$fun(x, y, env$env, outer.prod)
                }
                nrk <- 1
                irk <- irk.wk
                irk.wk <- irk.wk + nrk
                rk <- list(fun = rk.fun, env = rk.env)
            }
            if (type.wk == "tp") {
                par <- var.type[[vlist]][[2]]
                order <- par$order
                mesh <- par$mesh
                weight <- par$weight
                if (is.vector(x))
                  xdim <- 1
                else xdim <- dim(x)[2]
                phi.env <- mkphi.tp(xdim, order, mesh, weight)
                phi.fun <- function(x, nu = 1, env) {
                  env$fun(x, nu, env$env)
                }
                nphi <- choose(xdim + order - 1, xdim) - 1
                iphi <- iphi.wk
                iphi.wk <- iphi.wk + nphi
                phi <- list(fun = phi.fun, env = phi.env)
                rk.env <- mkrk.tp(xdim, order, mesh, weight)
                rk.fun <- function(x, y, nu = 1, env, outer.prod = FALSE) {
                  env$fun(x, y, env$env, outer.prod)
                }
                nrk <- 1
                irk <- irk.wk
```

```
                irk.wk <- irk.wk + nrk
                rk <- list(fun = rk.fun, env = rk.env)
            }
            if (type.wk == "custom") {
                par <- var.type[[vlist]][[2]]
                nphi <- par$nphi
                if (nphi > 0) {
                  phi.env <- par$mkphi(par$env)
                  phi.fun <- function(x, nu = 1, env) {
                    env$fun(x, nu, env$env)
                  }
                  iphi <- iphi.wk
                  iphi.wk <- iphi.wk + nphi
                  phi <- list(fun = phi.fun, env = phi.env)
                }
                rk.env <- par$mkrk(par$env)
                rk.fun <- function(x, y, nu = 1, env, outer.prod = FALSE) {
                  env$fun(x, y, env$env, outer.prod)
                }
                nrk <- 1
                irk <- irk.wk
                irk.wk <- irk.wk + nrk
                rk <- list(fun = rk.fun, env = rk.env)
            }
        }
        else {
            bin.fac <- n.phi <- phi.list <- rk.list <- NULL
            for (i in 1:dm) {
                type.wk <- var.type[[vlist[i]]][[1]]
                if (type.wk %in% c("nominal", "ordinal")) {
                  if (type.wk == "nominal")
                    rk.wk <- mkrk.nominal(levels(x[[i]]))
                  else rk.wk <- mkrk.ordinal(levels(x[[i]]))
                  phi.wk <- rk.wk
                  n.phi <- c(n.phi, 0)
                  bin.fac <- c(bin.fac, !(nlevels(x[[i]]) > 2))
                }
                if (type.wk == "cubic") {
                  range <- var.type[[vlist[i]]][[2]]
                  phi.wk <- mkphi.cubic(range)
                  n.phi <- c(n.phi, 1)
                  rk.wk <- mkrk.cubic(range)
                  bin.fac <- c(bin.fac, 0)
                }
                if (type.wk %in% c("cubic.per", "linear", "linear.per",
                  "sphere")) {
                  range <- var.type[[vlist[i]]][[2]]
                  n.phi <- c(n.phi, 0)
                  phi.wk <- NULL
                  if (type.wk == "cubic.per")
                    rk.wk <- mkrk.cubic.per(range)
                  if (type.wk == "linear")
                    rk.wk <- mkrk.linear(range)
                  if (type.wk == "linear.per")
                    rk.wk <- mkrk.linear.per(range)
                  if (type.wk == "sphere")
                    rk.wk <- mkrk.sphere(range)
                  bin.fac <- c(bin.fac, 0)
                }
                if (type.wk == "tp") {
                  par <- var.type[[vlist[i]]][[2]]
                  order <- par$order
                  mesh <- par$mesh
                  weight <- par$weight
                  if (is.vector(x[[i]]))
                    xdim <- 1
                  else xdim <- dim(x[[i]])[2]
```

```
          phi.wk <- mkphi.tp(xdim, order, mesh, weight)
          n.phi <- c(n.phi, choose(xdim + order - 1,
            xdim) - 1)
          rk.wk <- mkrk.tp(xdim, order, mesh, weight)
          bin.fac <- c(bin.fac, 0)
        }
        if (type.wk == "custom") {
          par <- var.type[[vlist[i]]][[2]]
          n.phi <- c(n.phi, par$nphi)
          if (par$nphi > 0)
            phi.wk <- par$mkphi(par$env)
          else phi.wk <- NULL
          rk.wk <- par$mkrk(par$env)
          bin.fac <- c(bin.fac, 0)
        }
        phi.list <- c(phi.list, list(phi.wk))
        rk.list <- c(rk.list, list(rk.wk))
    }
    if (!all(as.logical(n.phi + bin.fac)))
        nphi <- 0
    else {
        phi.env <- list(dim = dm, phi = phi.list, n.phi = n.phi,
          bin.fac = bin.fac)
        phi.fun <- function(x, nu = 1, env) {
          ind <- nu - 1
          z <- 1
          for (i in 1:env$dim) {
            if (env$bin.fac[i]) {
              wk <- as.factor(names(env$phi[[i]]$env$code)[1])
              z <- z * env$phi[[i]]$fun(x[[i]], wk, env$phi[[i]]$env)
            }
            else {
              code <- ind%%env$n.phi[i] + 1
              ind <- ind%/%env$n.phi[i]
              z <- z * env$phi[[i]]$fun(x[[i]], code,
                env$phi[[i]]$env)
            }
          }
          z
        }
        nphi <- prod(n.phi + bin.fac)
        iphi <- iphi.wk
        iphi.wk <- iphi.wk + nphi
        phi <- list(fun = phi.fun, env = phi.env)
    }
    rk.env <- list(dim = dm, n.phi = n.phi, nphi = nphi,
        phi = phi.list, rk = rk.list)
    rk.fun <- function(x, y, nu = 1, env, outer.prod = FALSE) {
        n.rk <- ifelse(env$n.phi, 2, 1)
        ind <- nu - (!env$nphi)
        z <- 1
        for (i in 1:env$dim) {
          code <- ind%%n.rk[i] + 1
          ind <- ind%/%n.rk[i]
          if (code == n.rk[i]) {
            z <- z * env$rk[[i]]$fun(x[[i]], y[[i]],
              env$rk[[i]]$env, outer.prod)
          }
          else {
            z.wk <- 0
            for (j in 1:env$n.phi[i]) {
              phix <- env$phi[[i]]$fun(x[[i]], j, env$phi[[i]]$env)
              phiy <- env$phi[[i]]$fun(y[[i]], j, env$phi[[i]]$env)
              if (outer.prod)
                z.wk <- z.wk + outer(phix, phiy)
              else z.wk <- z.wk + phix * phiy
            }
```

```
                z <- z * z.wk
              }
            }
            z
        }
        n.rk <- ifelse(n.phi, 2, 1)
        nrk <- prod(n.rk) - as.logical(nphi)
        irk <- irk.wk
        irk.wk <- irk.wk + nrk
        rk <- list(fun = rk.fun, env = rk.env)
    }
    term[[label]] <- list(vlist = vlist, iphi = iphi, nphi = nphi,
        phi = phi, irk = irk, nrk = nrk, rk = rk)
}
term
}
## modify the original "mkterm" function in "gss" package to perform pRKHS
unlockBinding("mkterm", as.environment("package:gss"))
assign("mkterm",mkterm,"package:gss")
##### Select markers for pRKHS #########
Bcoef <- NULL
for(j in 1:P){
    lmfit <- lm(y_train~x_train[,j])
    res <- try(c(Bcoef,summary(lmfit)$coef[2,1]),TRUE)
    if(class(res)=="try-error") next;
    Bcoef <- res;
}
pindex2 <- order(abs(Bcoef),decreasing=T)[1:maknum]
x_train.new <- x_train[,pindex2]            ## reduced train data matrix
x_test.new <- x_test[,pindex2]              ## reduced test data matrix
oldmean <- apply(x_train.new,2,mean);       ## Perform PCA
new.train <- t(t(x_train.new)-oldmean);
train.svd <- svd(new.train);
temp <- cumsum(train.svd$d^2)/sum(train.svd$d^2)
new.train <- new.train%*%train.svd$v;
new.test <- t(t(x_test.new)-oldmean)%*%train.svd$v
record <- NULL;
for(z in PV){
    #####  pRKHS-E ##########
    PC <- length(temp[temp<z])
    dta <- data.frame(cbind(y_train,new.train[,1:PC]))
    cos.inter <- NULL
    len <- dim(dta)[2]
    p.val <- NULL
    for(j in 2:len){
        x1 <- dta[,j]
        b<-ssanova(y_train~x1,data=dta)
        p.val <- rbind(p.val,c(summary(b,T)$cos[1,1],j))
        if(j==len) break;
        for(j1 in (j+1):len){
            x2 <- dta[,j1]
            res <- try(ssanova(y_train~x1*x2,data=dta),TRUE)
            if(class(res)=="try-error") next;
            sb <- summary(res,T)$cos
            temp3 <- sb[1,3]
            cos.inter <- rbind(cos.inter,cbind(temp3,j,j1))
        }
    }
    for (k in cosine){
        number1 <- length(cos.inter[,1][cos.inter[,1]>k])
        pindex1 <- try(order(cos.inter[,1],decreasing=T)[1:number1],TRUE)
        number2 <- length(p.val[,1][p.val[,1]>0.05])
        pindex2 <- order(p.val[,1],decreasing=T)[1:number2]
        vnam <- NULL
        for(i2 in pindex2)
            vnam <- c(vnam,paste("V",p.val[i2,2],sep=""))
        for(i in pindex1)
```

```
                vnam <- c(vnam,paste("V",cos.inter[i,2],":V",cos.inter[i,3],sep=""))
            fmla <- as.formula(paste("y_train~",paste(vnam,collapse="+")))
            res <- try(ssanova(fmla,data=dta),T)
            if(class(res)=="try-error"){
                record <- rbind(record,c(paste("fit1_",k,"_",maknum,"_PC_",z,seq=""),rep(NA,6)))}
            else{
                mst <- predict(res,res$mf,se.fit=T)
                new <- data.frame(cbind(y_test,new.test[,1:PC]))
                est <- predict(res,new[,-1],se.fit=T)
                cl <- cor(y_test,est$fit)                               ## Pearson correlation
                cl.sp <- cor(y_test,est$fit,method="spearman")          ## Spearman correlation
                cl.coef <- summary(lm(y_test~est$fit))$coef[2]
                record <- rbind(record,c(paste("fit1_",k,"_",maknum,"_PC_",z,seq=""),round(cl,3),
                            round(cl.coef,3),round(cl.sp,3)))
            }
        }
        ##### pRKHS-NE use no interations among PCs #########
        PC <- length(temp[temp<z])
        dta <- data.frame(cbind(y_train,new.train[,1:PC]))
        fit2 <- try(ssanova(y_train~.,data=dta),T)
        if(class(fit2)=="try-error"){
            record <- c(paste("fit2_",maknum,"_PC_",z),rep(NA,6))}
        else{
            mst <- predict(fit2,fit2$mf,se.fit=T)
            new <- data.frame(cbind(y_test,new.test[,1:PC]))
            est <- predict(fit2,new[,-1],se.fit=T)
            cl <- cor(y_test,est$fit)
            cl.sp <- cor(y_test,est$fit,method="spearman")
            cl.coef <- summary(lm(y_test~est$fit))$coef[2]
            record <- rbind(record,c(paste("fit2_",maknum,"_PC_",z),round(cl,3),
                        round(cl.coef,3),round(cl.sp,3)))
        }
    }
    return(record)
}

##################### Barley Data ########################
index <- read.table("index.txt",header=T)       ## Shared marker index between 2007 and 2008 data
dta2007 <- read.table("NDSU2007data.txt",header=T)
dta2008 <- read.table("NDSU2008data.txt",header=T)
traingen <- dta2007[,-(1:3)]
testgen <- dta2008[,-(1:3)]
traingene <- traingen[,index[,1]]
testgene <- testgen[,index[,2]]
X <- traingene
Y <- as.numeric(dta2007[,2])                              ## 2007 grain yield as training data set
n <- dim(X)[1]
vec1 <- apply(X,2,function(x){length(x[x==1])})
vec2 <- apply(X,2,function(x){length(x[x==0])})
p.vec <- (2*vec1+vec2)/(2*n)
frq.index <- p.vec<=.95 & p.vec>=.05      ## keep minor allele frequency larger than 0.05
snp <- X[,frq.index]
traingene <- traingene[,frq.index]
testgene <- testgene[,frq.index]
P <- ncol(snp)
########## Cross Validation of 2007 data #################
sindex <- sample(1:n)
q <- floor(n/10)
## set percent of variance explained by SPC ##
PV <- 0.7
## set cosine values to be tested ##
cosine <- c(0.3,0.25)
## set marker subset to be evaluated, in this case from 500 markers to full markers ##
maknum <- c(seq(from=500,to=1500,by=100),P)
record <- NULL
## 10 fold CV ##
for(r in 1:10){
```

```
    if(r==1){
        index <- sindex[1:(r*q)]
    }
    else if(r==10){
        index <- sindex[((r-1)*q+1):n]
    }
    else{
        index <- sindex[((r-1)*q+1):(r*q)]
    }
    y_train <- Y[-index]
    x_train <- snp[-index,]
    x_test <- snp[index,]
    y_test <- Y[index]
    ccc1 <- lapply(1:length(maknum),function(i,Red.RKHS,y_train,x_train,y_test,x_test,maknum,PV,
                cosine,P){Red.RKHS(y_train,x_train,y_test,x_test,maknum[i],PV,cosine,P)},
                Red.RKHS=Red.RKHS,y_train=y_train,x_train=x_train,y_test=y_test,x_test=x_test,
                maknum=maknum,PV=PV,cosine=cosine,P=P)
    for(k in 1:length(maknum))
        record <- rbind(record,ccc1[[k]])
    print(record)
}
################################################
#### Predict 2008 data ########################
y_train <- as.numeric(Y)
x_train <- traingene
P <- ncol(x_train)
x_test <- testgene
Y <- dta2008[,2]                           ## 2008 grain yield as testing set
y_test <- as.numeric(Y)
ccc1 <- lapply(1:length(maknum),function(i,Red.RKHS,y_train,x_train,y_test,x_test,maknum,PV,cosine,P){
              Red.RKHS(y_train,x_train,y_test,x_test,maknum[i],PV,cosine,P)},Red.RKHS=Red.RKHS,
              y_train=y_train,x_train=x_train,y_test=y_test,x_test=x_test,maknum=maknum,PV=PV,
              cosine=cosine,P=P)
## record has three columns: Pearson correlation, regression coefficient, Spearman correlation
for(k in 1:length(maknum))
    record <- rbind(record,ccc1[[k]])
###########################################
```
